# Implant removal associated complications after ESIN osteosynthesis in pediatric fractures

**DOI:** 10.1007/s00068-021-01763-4

**Published:** 2021-08-02

**Authors:** Justus Lieber, Markus Dietzel, Simon Scherer, Jürgen F. Schäfer, Hans-Joachim Kirschner, Jörg Fuchs

**Affiliations:** 1 Department of Pediatric Surgery and Pediatric Urology, University Children’s Hospital, Tübingen, Germany; 2 Department of Diagnostic Radiology, University Hospital, Hoppe-Seyler-Strasse 3, 72076 Tübingen, Germany

**Keywords:** Pediatric trauma, ESIN, TEN, Implant removal, Complication

## Abstract

**Purpose:**

ESIN (elastic stable intramedullary nailing) is considered the gold standard for various pediatric fractures. The aim of this study was to analyze the incidence and type of complications during or after TEN (titanium elastic nail) removal.

**Methods:**

A retrospective data analysis was performed. Metal removal associated complications and preoperative extraosseous length/outlet angle of TENs as possible causes of complications were assessed.

**Results:**

The complication rate in 384 TEN removals was 3.1% (*n* = 12). One major complication (rupture of M. extensor pollicis brevis) was documented. One refracture at the forearm occurred, however, remodeling prior TEN removal was completed. Ten minor complications were temporary or without irreversible restrictions (3 infections, 5 scaring/granuloma, 2 temporary paraesthesia).

In 38 cases (16 forearms, 10 femora, 9 humeri, 3 lower legs), intra-operative fluoroscopy had to be used to locate the implants. In patients with forearm fractures, extraosseous implant length was relatively shorter than in cases without fluoroscopy (*p* = 0.01), but outlet angle of TENs was not significantly different in these two groups (28.5° vs 25.6°). In patients with femur fractures, extraosseous implant length and outlet angle were tendentially shorter, respectively, lower, but this did not reach statistical significance.

**Conclusion:**

Removal of TENs after ESIN is a safe procedure with a low complication rate. Technically inaccurate TEN implantation makes removal more difficult and complicated. To prevent an untimely removal and patient discomfort, nail ends must be exactly positioned and cut. Intraoperative complications may be minimized with removal of TENs before signs of overgrowth.

**Evidence:**

Level III, retrospective.

## Introduction

Elastic stable intramedullary nailing (ESIN) is the gold standard for the treatment of pediatric diaphyseal and special metaphyseal fractures for more than 30 years [1]. A major benefit of this minimally invasive technique is the rapid castless postoperative mobilization. Also maintaining a high fracture stability and excellent axial alignment are realized. When bone healing and remodeling are completed, implants are being subsequently removed in a second operation. This is warranted to prevent chronic infections, interference of surrounding soft tissues, and impediments of fracture treatment later in life [2, 3]. For long, metal removal has been considered a standard procedure; however, it is very controversial whether implant removal in children free of material-associated complaints should routinely be performed. Several studies have discussed the pros and cons of implant removal in children showing that up to 60% of surgeons routinely remove implants after bone healing based on the surgeon's preferences, parental wishes or simply in general. Operation-related complications due to implant removal do also play a key role in this discussion [4, 5].

The aim of this study was to analyze the incidence and type of complications that occurred during or after the removal of TEN (titanium elastic nails) in children. Although ESIN and its associated metal removal is a daily routine procedure, the literature on this topic is still very sparse.

## Materials and methods

### Patients and ethical considerations

All patients undergoing ESIN osteosynthesis at our institution between January 2004 and December 2013 were retrospectively analyzed. The study was approved by the local Ethical Committee (number 704/2016BO2). Data on demographic characteristics, clinical background, indication for operation, intraoperative findings, and postoperative outcomes of the initial fracture treatment were collected from hospital records and stored on a computerized database. Indications, operation data, and complications at metal removal were also documented. Fractures were classified according to the AO Fracture and Dislocation Classification [6] including AO Pediatric Classification, AO Investigation and Documentation Group [7]. Complications were graded according to the classification proposed by Dindo and Clavien [8] and further classified as minor or major complications. Major complications were those that would require additional surgical intervention or resulted in permanent restriction for the patient (Dindo/Clavien grades III and IV). Data were acquired and processed according to the latest version of the “World Medical Association Declaration of Helsinki—Ethical Principles for Medical Research Involving Human Subjects”.

**Fig. 1:**
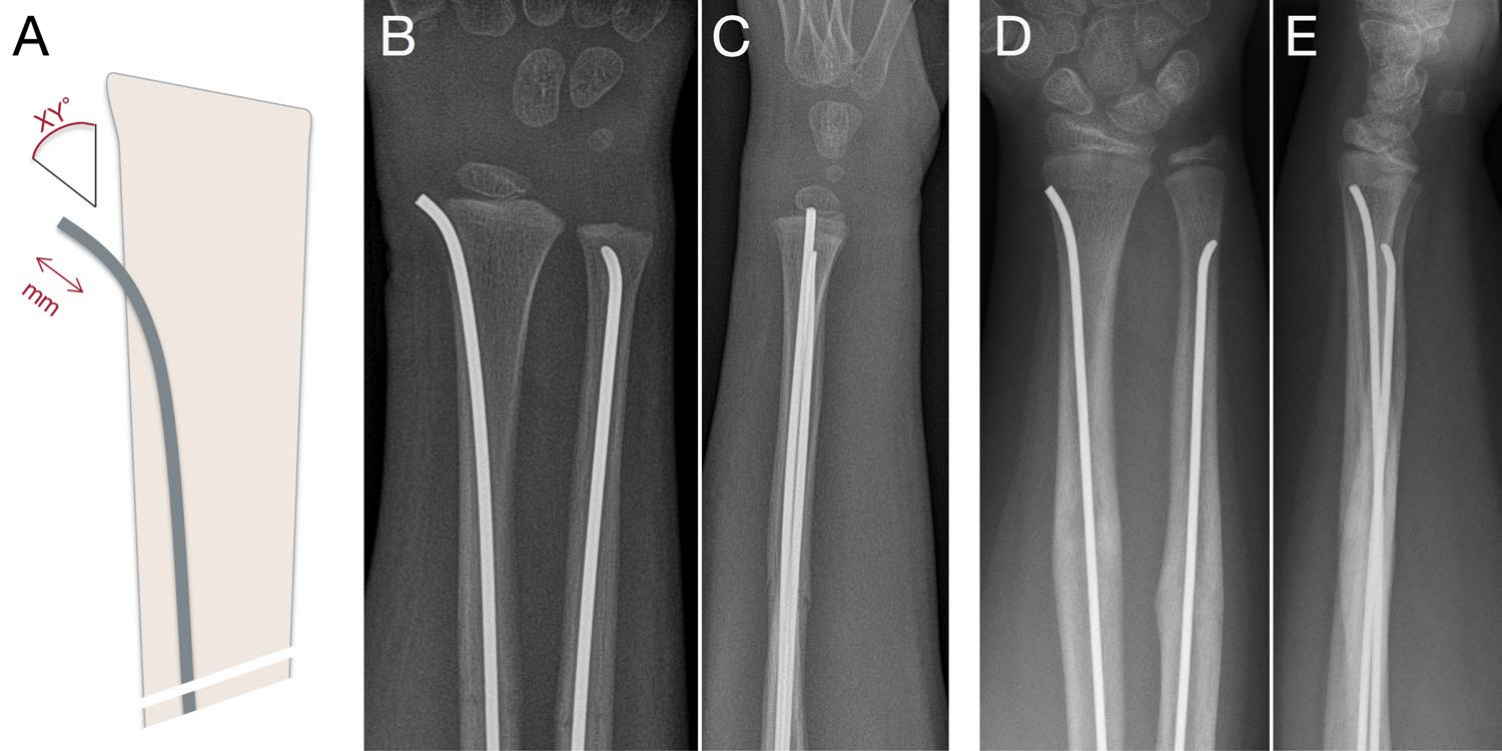
Measurements of extraosseous length and outlet angle of TENs at the distal radius (**A**). Finally positioned, the TEN should exceed the tendons, nor should cause a soft tissue problem (**B**, **C**: a.p. and lateral view). TENs should not be cut too short, because this may be followed by invasive and complicated removal (**D**, **E**)

TENs were implanted in such a manner (nail ends were situated outside the cortical bone and above tendons or fascias) that planned removal surgery after bone remodeling would be a minimal intervention. As a possible cause of complications, the extraosseous implant length (mm) and outlet angle (°) were assessed on X-rays (a.p.-projection) after fractures of the radius and femur (Fig. 1). In the lateral projection, it was ensured that TENs were implanted laterally in the bone to reduce mismeasurement in the a.p.-projection. Measurements were performed on the latest X-ray prior metal removal using a 17-inch dedicated workstation with a magnification of four times supervised by a pediatric radiologist. Mean values from three measurements were used.

### Statistics

Statistical analysis of extraosseous implant length and outlet angle was carried out using *t* test and correlation test with SPSS Statistics (www.ibm.com). *p* < 0.05 was considered statistically significant.

## Results

### Initial fracture treatment

A total of 432 patients underwent ESIN osteosynthesis within the 10-year period. The distribution of fractures is summarized in Table 1. Mean age of the patients was 7.2 years (1–15). Boys were more often affected than girls (287: 145); left and right sides were equally distributed (218: 214). Causes of injury were low-velocity falls (268), sport injuries (86), traffic injuries (47), polytrauma (18), and pathological fractures (13) on the basis of bone cysts, fibromas, and osteoporosis. Indication for reduction and osteosynthesis was intolerable age-dependent fracture dislocation, (358), instability per se (24) or during reduction (21), polytrauma (13) or others (16). The complication rate of initial fracture management was 5.2%, with a detailed description in Table 1. In 3 cases, early TEN removal became necessary because of skin-irritating nail ends.

**Table 1:** Distribution of fractures treated with ESIN and complications

Localisation	*n*	AO classification* (code)	%	Pathological fractures (*n*)	Complications (*n*)
Clavicle	1	1 diaphyseal (15.2A)	0.2		1 pseudarthrosis
Humerus	49	27 proximal (11-M/3.1 and 11-E/1.1) 7 diaphyseal (12-D/4.1 and 5.1) 15 supracondylar (13-M/3.1 II-IV)	11.3	4 bone cysts 1 non-ossified fibroma	4 secondary dislocations/instability 1 axial deviation < 10° 1 lesion of the ulnar nerve 1 persistence of bone cyst 1 temporary loss of sensibility
Ulna	24	16 diaphyseal (22u-D/4.1) 8 Monteggia (22u-D/6.1)	5.6		
Radius	19	10 diaphyseal (22r-D/5.1) 9 radial neck (21r-E and 21r-M)	4.4		
Ulna and radius	207	207 diaphyseal (22-D1.1–5.1)	47.9	1 bone cyst	2 secondary dislocations/instability 2 temporary losses of sensibility
Metacarpale	7	2 proximal (77._.1) 5 distal (77._.3)	1.6		
Femur	68	68 diaphyseal (32-D4.1 and 5.1)	15.8	2 bone cysts 2 osteoporotic bones	9 secondary dislocations/instability 1 refracture (nails in situ) 1 effusion/pain at nail entry 3 premature metal removals
Lower leg	55	2 proximal (41-M/3.1) 23 diaphyseal (42-D/4.1 and 5.1) 30 distal (43-M/3.1)	12.6	2 ossifying fibromas 1 bone cyst	2 refractures (nails in situ) 2 infections/osteomyelitis 1 secondary dislocation/instability 1 compartment syndrome 1 hematoma
Metatarsale	2	2 subcapital (87._.3)	0.5		
Total	432		100	13	34

*AO Fracture and Dislocation Classification as well as AO Pediatric Classification Group and AO Investigation and Documentation Group

### Implant removal

Implant removal was performed in our Department in 384 of the 432 cases (89%). The operation was performed as a day case surgery procedure in 92% of the cases; thirty-one patients had an overnight stay in the hospital because of a revision procedure (*n* = 19), an underlying disease, such as congenital heart defects or diabetes (*n* = 7), an additional operative procedure, e.g. cystoscopy or neurosurgical operation (*n* = 4), or due to pain requiring i.v.-analgesia (*n* = 1). The duration of metal remaining in situ accounted for a mean of 136 days (1–1015). The average operation time for metal removal was 33 min (5–99). Operation data and complications are shown in Table 2. Complications occurred in 3.1% of the cases during or after TEN removal. According to the used classification, only one major complication was observed. This consisted of a rupture of the tendon of the M. extensor pollicis brevis. The injury was recognized intraoperatively and the reconstruction was performed during the same anesthesia with subsequent immobilization in a plaster splint for 3 weeks. After mobilization, no restriction of movement was found at last presentation. In one patient, a refracture at the forearm occurred, however, remodeling prior to TEN removal was completed. Ten minor complications were temporary or without irreversible restrictions: superficial/deep infections (*n* = 3), hypertrophic scarring and/or granuloma formation (*n* = 5), and temporary paraesthesia/hyposensibility at the thumb as a sign of damage to the superficial radial nerve (*n* = 2).

**Table 2:** Data and results of TEN removal

Localisation	*n*	Metal in situ *d* (range)	Duration of MR min (range)	X-ray (*n*)	X-ray min (range)	Complications (*n*)	Complications (according to Dindo/Clavien^7^)
Clavicle	1	432		*–*		*–*	
Humerus	49	150 (1–631)	28 (7–87)	9	18 (2–64)	3 hypertrophic scarring 1 superficial wound infection	I I
Forearm	205	125 (9–792)	29 (7–126)	16	19 (2–120)	2 temporary loss of sensibility 1 rupture of tendon (M. ext. poll brevis) 1 refracture* 1 hypertrophic scarring 1 superficial wound infection 1 subcutaneous granuloma	I IIIb IIIb I I I
Metacarpale	7	92 (35–149)		*–*	*–*	*–*	
Femur	66	139 (1–347)	33 (5–99)	10	39 (2–124)		
Lower leg	54	176 (6–1015)	36 (5–85)	3	16 (3–37)	1 deep wound infection	II
Metatarsale	2	72 (64–81)		*–*	*–*	*–*	
Total	384	136 (1–1015)		38		12	

*Refracture occurred despite remodeling had completed; therefore, it may not be considered a complication of metal removal

In 38 of 384 cases (10%) intraoperative fluoroscopy was used to detect implants, but the radiation dose was not available in most of the cases. The extraosseous implant length and outlet angle for the exemplary locations of the distal radius and distal femur are displayed in Table 3. Only cases that received 2 TENs inserted with the same technique were considered (forearm: antegrade nailing of the ulna, retrograde nailing of the radius; femur: retrograde nailing). Results are separately shown for those cases with or without intraoperative fluoroscopy. In summary, significantly more often fluoroscopy was required at the distal radius to locate the TEN if it had been shortened too much (*p* = 0.01). For the distal femur, this applied only to the length of the medially inserted nail (*p* = 0.03). There was no significant difference in length of the lateral nail (*p* = 0.44) or outlet angle (medial *p* = 0.30; lateral *p* = 0.10).

**Table 3:** Extraosseous length and outlet angle of TENs

	*n*	Extraosseous TEN length (mm)				Extraosseous TEN outlet angle (°)			
Distal radius									
Total	170	7.3				25.9			
MR without X-ray	154	7.5		*p = 0.01		28.5		*p* = 0.47	
MR with X-ray	16	4.9				25.6			
Distal femur		Medial TEN		Lateral TEN		Medial TEN		Lateral TEN	
Total	49	14.8		15.0		20		27	
MR without X-ray	39	16.2	*p = 0.03	15.3	*p* = 0.44	22	*p* = 0.30	28	*p* = 0.10
MR with X-ray	10	9.8		13.6		16		21	

Results at the distal radius and distal femur

*MR* metal removal, *TEN* titanium elastic nail

*Significant

## Discussion

The main finding of this study is a low complication rate of 3.1% (*n* = 12/384) during or after TEN removal in children. If only major complications are addressed, the complication rate drops to 0.26% (*n* = 1/388). This is in rough accordance with some sporadic studies in the literature focusing on TEN removal and reporting complication rates from 3.5 to 7% [9–11].

In general, there is no evidence in the current literature to support or to refute the routine removal of orthopedic implants in asymptomatic children [12]. Potential benefits of metal removal include prevention of biological and functional sequelae such as malignancy, infection, inflammation, and ease of later reconstructive or prosthetic surgery. However, all of these are very infrequent and without compelling evidence for causation. Also, the stated risks are limited to anecdotes and expert opinions in addition to literature reviews [2, 13, 14]. In symptomatic patients, premature metal removal is undoubtedly justified, e.g. if painful irritation of soft tissues or restriction of movement due to excessively long implant ends occur [14]. The results of the present study, therefore, lead to the conclusion that technically inadequate implant positioning both aggravates patient complaints and makes metal removal significantly more difficult and complicated. If implants are shortened too much or lie too close to the bone, the operation time for metal removal may be prolonged, and additional X-ray becomes required more often to locate the implants (Table 3). In these cases, metal removal is more invasive and increases the risk of injury to nearby structures. Consequently, the surgeon has to find a compromise between stability demands and patients’ discomfort when performing an ESIN osteosynthesis for pediatric extremity fractures.

**Fig. 2:**
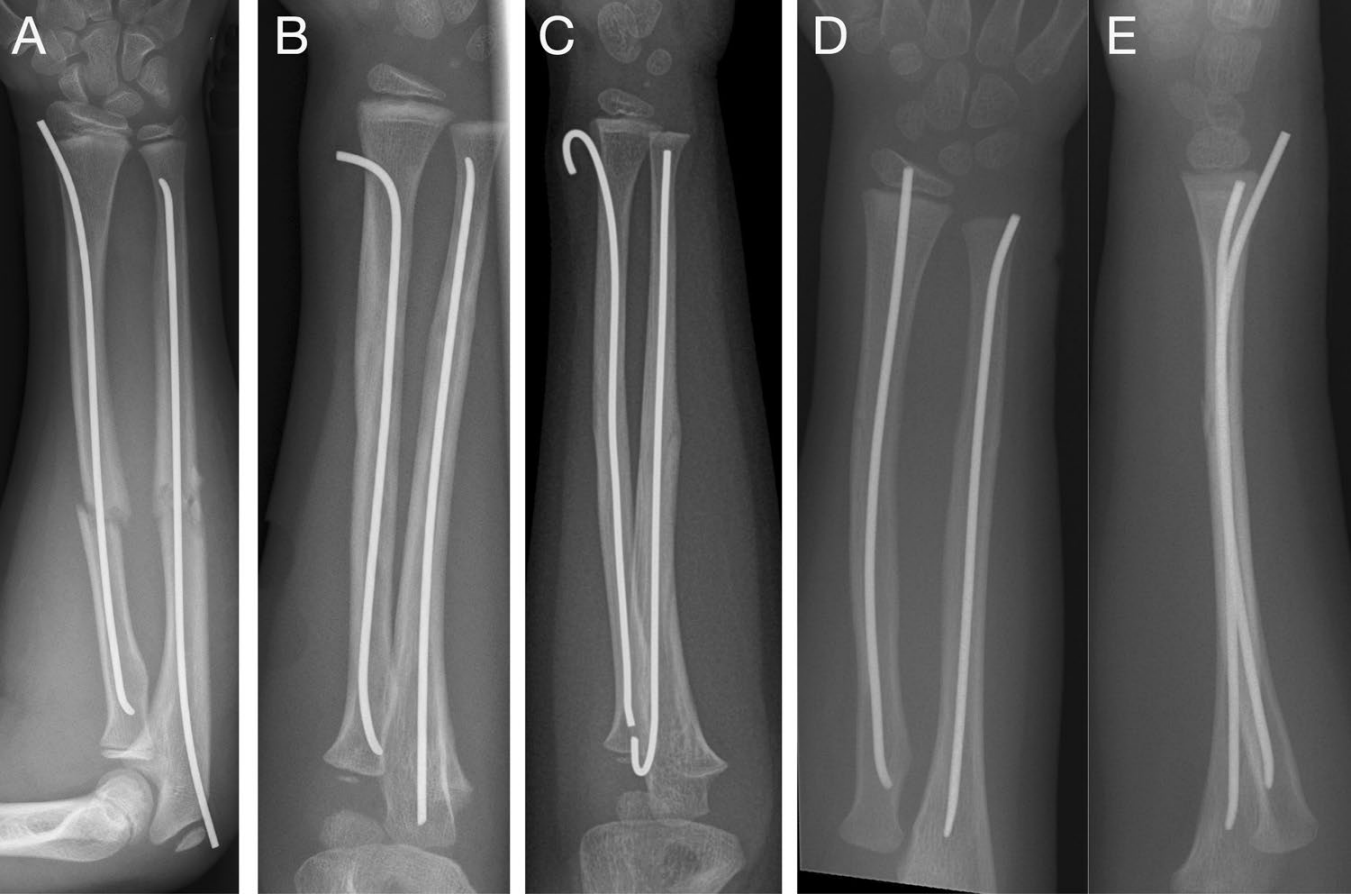
Ideally cut TEN implanted at the distal lateral radius (**A**), and distal dorsal radius (**D**, **E**). For better retrieval during metal removal, partial bending (**B**) or complete bending (**C**) of the nail are performed

**Fig. 3:**
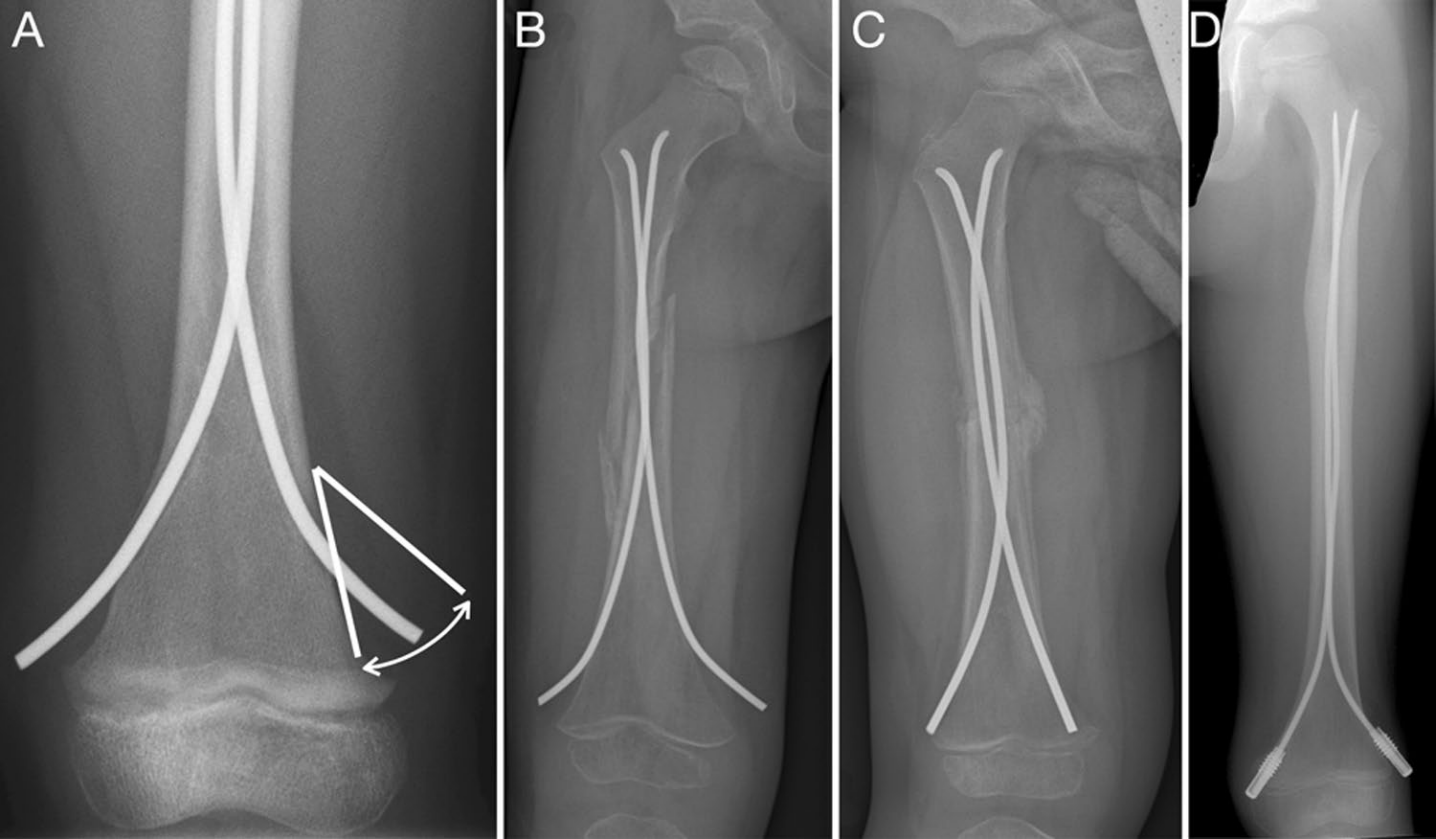
Measurements of extraosseous length and outlet angle of TENs at the distal femur (**A**). Example of sufficiently long TEN ends causing no discomfort in the patient and allowing an easy retrieval without need for fluoroscopy (**B**). Case in which the too shortly cut TENs required fluoroscopy and a prolonged operation time for retrieval (**C**). Additional tools such as end caps save the soft tissue and ease the metal removal (**D**)

As there are many locations to apply an ESIN osteosynthesis today, this provides different potential for complications during implant removal due to differences in soft tissue coverage or exposure of nail ends. The incidence and use of operative treatment of pediatric forearm fractures have increased since the 1980s [15, 16]. Therefore ESIN is increasingly used and described as the undisputed gold standard with various insertion sites at the distal forearm to handle the various fracture types. Sufficiently large incisions and proper instruments for smooth cutting of nails prevent primary and late injury to nerves (e.g. nervus radialis superficialis at the lateral distal radius) or tendons (e.g. extensor pollicis longus at the dorsal distal radius), and threat of soft tissue perforation [17]. For better retrieval during metal removal, partial bending or complete bending of the nails is observed (Fig. 2) [18], through which the 3-point support is questionable in its biomechanical quality. In addition, bending of nail ends may constrict or overstretch soft tissue structures. Even a novel surgical technique has been described by Gautam et al. using a metallic suction cannula to aid elastic nail removal [19]. Other authors report ultrasound scanning to help surgeons to locate the buried unpalpable metalwork and marking the tract by injection of methylene blue dye to ease removal [20]. At the femur, additional tools such as end caps or nail-locking screws can increase stability and prevent telescoping, as well as protect the soft tissue and simplify metal removal (Fig. 3) [21].

For all shaft fractures treated with ESIN, metal removal should only be performed when remodeling is completed to reduce the risk of refracture [9, 11]. Lascombes and coworkers suggest no nail removal until 1 year after surgery [22]. In our series, there was only 1 refracture on the forearm, whereas the rates reported in the literature range between 4 and 8% [23, 24]. To date, numerous risk factors for refractures of forearms have been analyzed. Male gender and the lower third location of the original fracture have been identified as respective risk factors [25]. However, if TENs are left in the medullary canal beyond the time of remodeling, late metal removal has been described to be more difficult and invasive or even impossible. There are also reports on the surface microtopography of nails to have a significant effect upon the force required for removal. Nails made of titanium–aluminum–niobium (TAN) promote strong bone on-growth and require higher pull-out forces compared to nails made of stainless steel [26, 27]. As we use TAN nails because of their excellent biocompatibility and mechanical properties, we cannot comment on this topic. However, in our perception, the forces required to remove implants are of less importance compared to an easy accessibility of the implants. Refractures or additional fractures with implants in situ are certainly more common in individual cases with osteoporotic or unstable bone structure on the basis of osteogenesis, bone cysts or spastic patients, where the metal is left for other reasons [14].

The actual complication rate at/after metal removal is very low in the present group; no complications at all have occurred at the femur, metacarpal and metatarsal. Higher complication rates in the upper extremities may be due to their higher fracture incidence, accounting for approximately 3/4 of all pediatric long bone fractures [28]. Probably even more decisive is the fact that the predilection sites for complications caused by a high density of important structures (e.g. tendons and nerves) at the TEN implantation sites are located in the upper extremities. This is especially true for the distal radius and the distal humerus, but of less relevance at the distal femur and proximal tibia. Overall, the complication rates in this cohort are comparable to those reported in the literature [9–11]. Nevertheless, this is—to our best knowledge—the first study to provide data on TEN implantation quality in correlation with the occurrence of complications on removal. This study has some limitations which have to be addressed: a limitation of our study is the retrospective design. Children, who experienced complaints or a refracture, may also have presented elsewhere and were thus lost to follow-up. In addition, the measurement of the implant ends protruding from the bone is imprecise in that a two dimensionality is found in the X-ray image, but in reality, a three dimensionality is present. To avoid such imprecise measurements in future studies, intraoperative fluoroscopy at the time of metal removal with an accurate adjustment of the projection could be an option. However, the detailed processing of the patient collective also with regard to primary care confirms that a clinically relevant group has been analyzed. Even though evaluated retrospectively, the data of this study are important in such a way that the complication rate reflects potential additional anesthesia and interventions as well as medical costs. Pins and K-wires can be removed in an outpatient situation under local anesthesia, but this does not apply to TENs. On the other hand, ESIN osteosynthesis for shaft fractures is unrivalled among all procedures, as it is the most biological technique. It is significantly less invasive compared e.g. with plate osteosynthesis, which has equally high complication rates for metal removal, or equally high refracture rates [29, 30].

In the future, resorbable implants will increasingly be used in pediatric traumatology, thus making metal removal unnecessary [31]. This also applies to resorbable ESIN, which are already being used in patients [32]. However, the stability of the BIN (bioabsorbable intramedullary nails) is still low, and additional plaster immobilization is necessary. This, in turn, does not compete with titanium TENs, which provide immediate free mobilization and early weight load. A second operative procedure for metal removal currently compensates for the advantages of the conventional ESIN method until a proper resorbable alternative has been found and established.

## Conclusion

In conclusion, removal of TENs in children after ESIN treatment is a safe procedure with a low complication rate. Technically inaccurate implant insertion makes metal removal more difficult and complicated, and may also result in patient discomfort. To prevent an untimely removal, nail ends must be exactly positioned and cut. Intraoperative complications may be minimized with the removal of TENs before signs of overgrowth.

## Data Availability

The datasets analyzed during the current work are available from the corresponding author upon reasonable request.
